# Calcium Carbonate@silica Composite with Superhydrophobic Properties

**DOI:** 10.3390/molecules26237180

**Published:** 2021-11-26

**Authors:** Yitong Ma, Pei Tian, Malayphone Bounmyxay, Yiwen Zeng, Nong Wang

**Affiliations:** 1 School of Chemistry and Chemical Engineering, Lanzhou Jiaotong University, Lanzhou 730070, China; mayitong0822@163.com (Y.M.); tienpei_977@163.com (P.T.); jingxiaolian012@163.com (M.B.); 2 Guangxi Key Laboratory of Calcium Carbonate Resources Comprehensive Utilization, College of Materials and Chemical Engineering, Hezhou University, Hezhou 542899, China

**Keywords:** composite material, roughness, hydrophobicity, contact angle

## Abstract

In this paper, spherical calcium carbonate particles were prepared by using CaCl_2_ aqueous solution + NH_3_·H_2_O + polyoxyethylene octyl phenol ether-10 (OP-10) + n-butyl alcohol + cyclohexane inverse micro emulsion system. Then, nanoscale spherical silica was deposited on the surface of micron calcium carbonate by Stöber method to form the composite material. Scanning electron microscope (SEM), X-ray powder diffraction (XRD), Fourier transform infrared spectroscopy (FTIR), Thermogravimetric analysis (TGA) and X-ray photoelectron spectroscopy (XPS) were used to characterize the morphology and structure of the composite material. It is found that the surface of the composite material has a micro-nano complex structure similar to the surface of a “lotus leaf”, making the composite material show hydrophobicity. The contact angle of the cubic calcium carbonate, spherical calcium carbonate and CaCO_3_@SiO_2_ composite material were measured. They were 51.6°, 73.5°, and 76.8°, respectively. After modification with stearic acid, the contact angle of cubic and spherical CaCO_3_ were 127.1° and 136.1°, respectively, while the contact angle of CaCO_3_@SiO_2_ composite was 151.3°. These results showed that CaCO_3_@SiO_2_ composite had good superhydrophobicity, and the influence of material roughness on its hydrophobicity was investigated using the Cassie model theory.

## 1. Introduction

Over thousands of years of natural selection, living organisms, including all plants and animals on our earth, have become evolutionarily optimized functional systems. One of their most fascinating properties is their ability of self-cleaning, which means that their surfaces can repel contaminants such as solid particles, organic liquids, and biological contaminants by the action of rolling-off water drops. Due to the lotus leaf being used as the typical representative, this kind of hydrophobicity or self-cleaning property has been known as the “lotus effect” [1,2,3,4,5,6,7,8]. In 1998, after Barthlott described lotus leaf self-cleaning behavior, the lotus leaf effect attracted wide attention in the study of biological surfaces, and many researchers focused on the preparation of various self-cleaning materials [9,10,11].

The contact and sliding angle of a drop are a quantitative measure of self-cleaning behavior. It is considered that both hydrophobicity and low contact angle hysteresis are necessary for self-cleaning surfaces [12,13,14]. The contact angle hysteresis is also related to the roughness and surface tension of the material. In general, in order to make a superhydrophobic surface (water contact angle > 150°), the nanometer scale roughness and hydrophobic surface (smooth surface contact angle > 90°) are two basic requirements [15]. 

Calcium carbonate is a kind of important inorganic chemical raw materials. Due to its many advantages such as a rich material source, simple working process, high product quality, low cost, etc., micro and nano calcium carbonate powder have been widely used in the paper making [16,17], paint, rubber [18], and adhesives industry [19]. In addition, due to their good stability, biocompatibility and biodegradability, calcium carbonate can be used as drug or gene carrier in biomedical fields and substrate for the manufacturing of mixed materials with different functions [20,21]. 

The finer the surface of calcium carbonate, the higher the surface energy and the easier it is to self-agglomerate in polar solvents. Its surface is hydrophilic and oleophobic, and it is therefore not easy to bind to organisms with low surface energy and it is difficult to disperse in polymers. By modifying the surface of calcium carbonate powder, its surface energy can be reduced, its dispersibility can be improved, and its surface can be changed from hydrophilic to hydrophobic [22].

As a kind of multifunctional material, silica is used in many fields, such as in microelectronic devices, optical fiber, and can also be used as a chromatographic column packing and catalyst carrier [23,24,25]. Because its properties are very stable and its surface can be modified easily with appropriate heat treatment or chemical treatment, which are responsible for the physical and chemical properties of silica, it thus makes these materials very attractive for academic research and technical applications [26,27,28,29,30,31]. Nano-silica is the most widely used inorganic nonmetallic nanomaterials at present. There are different bonded hydroxyl groups on the surface of nano-silica, which mainly have three forms: (1) isolated silanols, (2) vicinal silanols, and (3) geminal silanols. These groups have strong water absorption and are prone to agglomeration [32].

Composite materials are composed of two or more materials with different properties by physical or chemical methods, which have a series of excellent characteristics, such as good bearing capacity, excellent mechanical properties, strong vibration reduction, wear resistance, heat resistance, and material design, etc. Various materials have complementary and synergistic effects in performance, so that the composite material’s comprehensive properties are better than the original constituent materials and meet different requirements [33,34,35]. So, if the nano scale silica is deposited with calcium carbonate surface to form a composite material, the overall roughness of the material can be increased [36]. 

The reversed-phase microemulsion method is a new material preparation method developed in recent years [37,38]. It is an oil-in-water (or water-in-oil) dispersion system composed of an oil phase, water phase, and a surfactant. The tiny “pool” in the inverting microemulsion is surrounded by a monolayer composed of surfactants and co-surfactants. Because of the special properties of the surfactant, the size of the “pool” can be controlled by adjusting the molar ratio of the surfactant. At present, the method of preparing nanomaterials using microemulsion as the template has been widely used in the preparation of composite catalysts, semiconductors, super-conductors, and magnetic nanoparticles [39,40]. This method of preparing micron or nano materials is easy to operate and does not need high temperature calcination. The size can be adjusted by changing its composition [41].

The wettability of a solid surface can be expressed by the contact angle (θ) of water droplets on the solid surface. If 90° < θ < 150°, the water droplets cannot easily infiltrate the solid surface, indicating that the solid surface is hydrophobic. If θ > 150°, the water droplets cannot infiltrate the solid surface, indicating that the solid surface is superhydrophobic. The free energy of the solid surface and the microstructure determine the wettability simultaneously [42].

**Figure 1:**
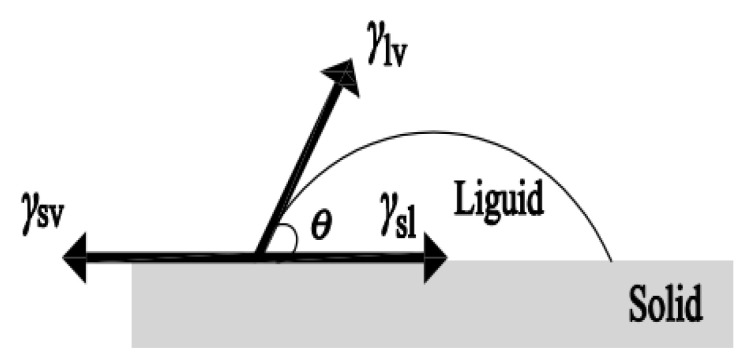
Standard vapor-solid-liquid three-phase interface.

As shown in Figure 1, the contact angle θ of liquid droplets on the solid surface is the angle between the solid-liquid interface and the tangent line of gas-liquid interface from the intersection point of gas liquid solid three phase [43]. Young’s equation is the relationship between the free energies of solid-gas, solid-liquid, and liquid-gas interfaces *γ*_sv_, *γ*_sl_, *γ*_lv_ and the contact angle *θ*, which is also known as the wetting equation, and is expressed as:

*γ*_sv_ − *γ*_sl_ = *γ*_lv_ cos*θ.* (1)

Cassie believed that the contact of liquid drops on the rough surface was a kind of composite contact. Droplets on the hydrophobic surface could not fill the grooves on the rough surface, and there was trapped air under the droplet in the grooves. Therefore, the apparent solid-liquid contact was actually composed of solid-liquid and solid-gas contact. From the perspective of thermodynamics, at equilibrium, the apparent contact angle of the rough surface *θ*_r_ has the following relationship with the intrinsic contact angle of smooth surface *θ*_s_:

cos*θ*_r_ = *f* (1 + cos*θ*_s_) − 1, (2)

where *f* is the ratio of the apparent contact area to the raised solid area in the composite contact surface (*f* < 1). *r* = 1/*f* is defined as the roughness of the material surface. So, the Cassie equation has the following relationship [44,45]:

cos*θ*_r_ = [(1 + cos*θ*_s_)/*r*] − 1. (3)

**Figure 2:**
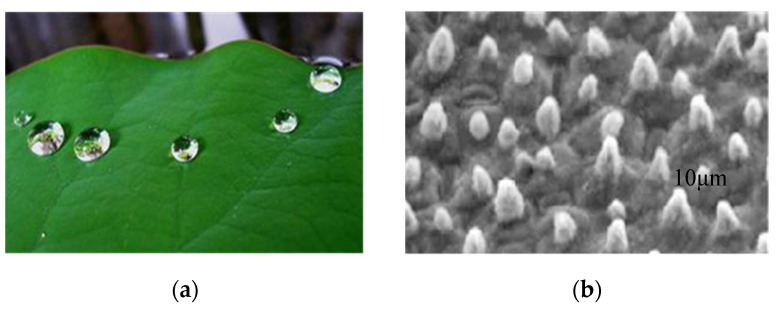
(**a**) Typical digital photographs of superhydrophobic lotus leaf and water droplet on the lotus leaf. (**b**) Low magnification scanning electron microscope (SEM) image of the surface structures on the lotus leaf.

According to the electron microscope image, a large number of micron-sized waxy micropapillae are distributed on the surface of lotus leaves, and a large number of nanoscale bulge structures are distributed on each micropapillae, as shown in the Figure 2 [46]. Such micron-nano composite structure results in a low contact area and a large contact angle between water droplets and the surface of the lotus leaf.

In this paper, composites similar to the surface structure of lotus leaves were prepared, and the influence of surface roughness on hydrophobicity was studied. Micron-sized spherical calcium carbonate was prepared by W/O microemulsion system with polyoxyethylene octyl phenol ether-10 (OP-10) as the surfactant [47,48], n-butanol as the co-surfactant, and cyclohexane as the oil phase. Nano spherical silica was prepared by the Stöber method [49,50,51], which was loaded on micron-sized spherical calcium carbonate. After being modified by stearic acid, the coating prepared with the composite material resembled the “lotus effect”.

## 2. Materials and Methods

### 2.1. Materials

A total of 25% ammonia solution and calcium chloride were supplied by Tianjin Damao Chemical Factory, China. OP-10 and cyclohexane was provided by Shandong Shuangshuang Chemical Factory, China. N-butyl alcohol were purchased from Shanghai Ron reagent, China. Tetraethyl orthosilicate (TEOS) and n-hexane were purchased from Shanghai Zhongqin Chemical reagent, China. Stearic acid and isooctyl tetraethoxy silane (ITES) were supplied by Laiyang Chemical Factory, Laiyang, China. Distilled water was produced by our laboratory and was applied for all reactions and treatment processes. All the chemicals were analytical grade and used without further purification.

### 2.2. Preparation Method

Spherical calcium carbonate: 0.5 mol/L calcium chloride aqueous solution was prepared with ammonia water and calcium chloride. Then, we mixed the aqueous solution, cyclohexane, n-butyl alcohol, and OP-10 to prepare a stable inverse microemulsion. The volume ratio of the surfactant, co-surfactant, oil phase, and water phase were 15:15:50:4. The CO_2_ gas was introduced into the microemulsion at the rate of 300 mL/min. With the continuous penetration of CO_2_, the microemulsion began to become turbid, and micron scale spherical calcium carbonate was formed. During the precipitation, the solution was continuously stirred at a constant rate of 650 r/min. The yield of the final product was 0.19 g.

CaCO_3_@SiO_2_ composite material: The nano scale silica was generated by TEOS, ethanol, and ammonia. A total of 500 μL TEOS was diluted by 2 mL ammonia and 5 mL ethanol. After stirring for 2 h, 250 μL of ITES was added to the organic moieties incorporation by condensation reaction of TEOS. It was mixed with the turbid micron scale spherical calcium carbonate solution mentioned above and stirred in a constant temperature shaker at 70 °C for 3 h. Then, it was dried in a vacuum drying oven at 100 °C for 24 h to ensure that the silica was successfully loaded on the calcium carbonate. Finally, it was immersed in n-hexane solution of stearic acid for 1 h and dried in an oven at 70 °C. The yield of the final product was 0.31 g. 

Cubic calcium carbonate: As a contrast, the cubic calcium carbonate was prepared. A total of 50 mL 0.5 mol/L calcium chloride aqueous solution, 1.1 mL OP-10, and 9 mL ethyl alcohol were mixed, then we let the solution stand for at least 48 h in the air. Micron scale cubic calcium carbonate particles were formed. The yield of the final product was 2.3 g.

### 2.3. Surface Treatment

A total of 20 g of dry powder of the materials mentioned above were well dispersed in 45 mL 0.5 mol/L stearic acid n-hexane solution. The suspension was continually stirred for 0.5 h. Then, the mixture was oven-dried at 70 °C. A double pressure sensitive adhesive tape with one side sticking on a glass slice was used as the substrate. The modified materials were spread on the surface of the adhesive tape and gently pressed with a glass cover. After pressing, the glass was flushed with air under pressure to remove free materials. This process, including powder spreading, pressing, and air flushing were repeated several times until the adhesive tape was fully covered by material particles. 

### 2.4. Equipment and Characterization

The size and morphology of the composite material powder samples were analyzed by scanning electron microscopy (SEM; JSM-6100, JEOL Ltd., Tokyo, Japan). Infrared spectroscopy was recorded by a Fourier transform infrared spectrometer (FTIR; EXUS–470, Nicolet, Tokyo, Japan). The crystal structure types were analyzed by powder X-ray diffraction (XRD; RINT-1000, Rigaku, Tokyo, Japan). The stability was studied by thermogravimetric analysis (TGA; TGA55, American TA Instrument Company, Hialeah, FL, USA). The contact angle was measured by a video optical contact angle tester (CA; OCA25, Eastern-Dataphy, Germany). The elements of the material were further analyzed with X-ray photoelectron spectroscopy (XPS; AXIS Supra, Kratos, Manchester, UK). All the glassware and storage bottles were immersed in hydrochloric acid overnight, then rinsed thoroughly with distilled water.

## 3. Results and Discussion

### 3.1. Morphology

**Figure 3:**
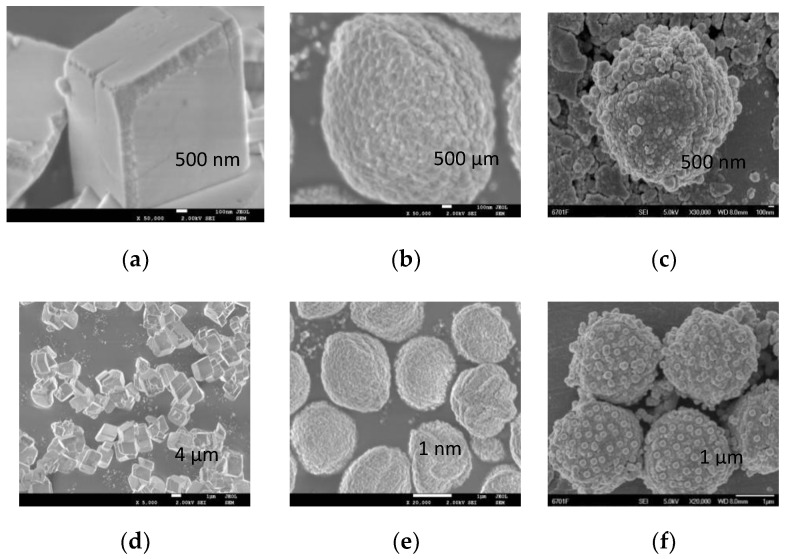
SEM image of cubic calcium carbonate (**a**,**b**), spherical calcium carbonate (**c**,**d**) and CaCO_3_@SiO_2_ composite material (**e**,**f**). CaCO_3_@SiO_2_ composite materials were formed by loading nanosized silica onto spherical calcium carbonate.

Figure 3 are the scanning electron microscope photos of different material types. Figure 3a,b shows the cubic calcium carbonate particles, Figure 3c,d shows the spherical calcium carbonate particles prepared by the reverse microemulsion method, Figure 3e,f shows the composite material obtained by depositing nanoscale spherical silica on the surface of micron calcium carbonate. It can be seen from Figure 3a,b that the surface of the cubic calcium carbonate is smooth without any bumps, while the spherical calcium carbonate in Figure 3c,d is slightly concave and convex. The surface roughness of the composite material in Figure 3e,f is greatly increased due to the overall surface heterogeneity caused by the presence of spherical silica.

### 3.2. Structure and Crystal Type

**Figure 4:**
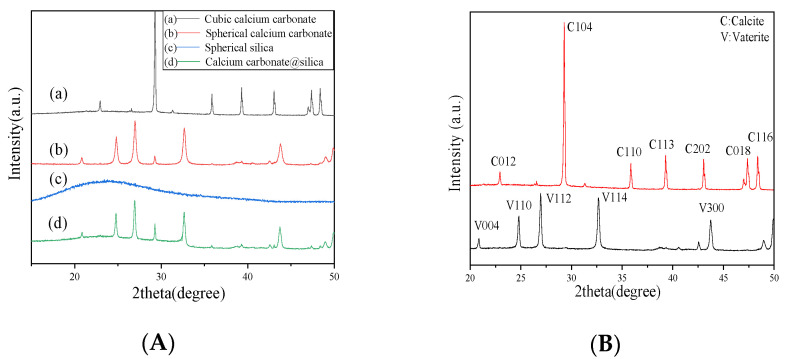
(**A**) X-ray diffraction pattern of different materials (a) cubic calcium carbonate, (b) spherical calcium carbonate, (c) spherical silica, and (d) CaCO_3_@SiO_2_ composite material. The black, red, blue, and green stick patterns in the XRD panels correspond to the (a), (b), (c), and (d). (**B**) The standard X-ray diffraction pattern of vaterite and calcite. The black and red stick patterns in the XRD panels correspond to the vaterite and calcite, respectively.

Figure 4 shows the X-ray diffraction pattern of different material types. Vaterite and calcite are the two main crystal forms of calcium carbonate. The standard peaks of calcite crystal are consistent with the PDF#47-1743 [52]. It can be found that the diffraction peaks of cubic calcium carbonate (Figure 4a) appear at 2*θ* of 22.90°, 29.21°, 35.84°, 39.40°, 43.75°, 47.27°, and 48.94°. This is the same as the standard X-ray diffraction pattern for calcite, indicating that the prepared cubic micron calcium carbonate crystal form is mainly calcite. The diffraction peaks of spherical calcium carbonate (Figure 4b) appear at 20.81°, 24.72°, 26.93°, 29.35, 32.60°, 43.83°, 48.92°, and 49.97°. The diffraction peaks at 20.8°, 24.7°, 26.9°, 32.6°, 43.8°, 48.9°, and 49.97° agree with the standard X-ray diffraction pattern for vaterite [53], the diffraction peaks at 29.35° agree with the standard X-ray diffraction pattern for calcite, indicating that the spherical calcium carbonate mainly has vaterite [54]. Figure 4c shows the X-ray diffraction pattern of silica, the intense broad peak at 2*θ* = 24° represents the amorphous silica due to the smaller particle size effect and incomplete inner structure of the spherical nanoparticles [55]. Figure 4d shows the X-ray diffraction pattern of composite material. Compared to Figure 4b,c, Figure 4d can be considered as a superposition of the Figure 4b,c spectrum, indicating that the composite material is composed with a large amount of vaterite calcium carbonate, a small amount of calcite calcium carbonate, and some amorphous silica. 

**Figure 5:**
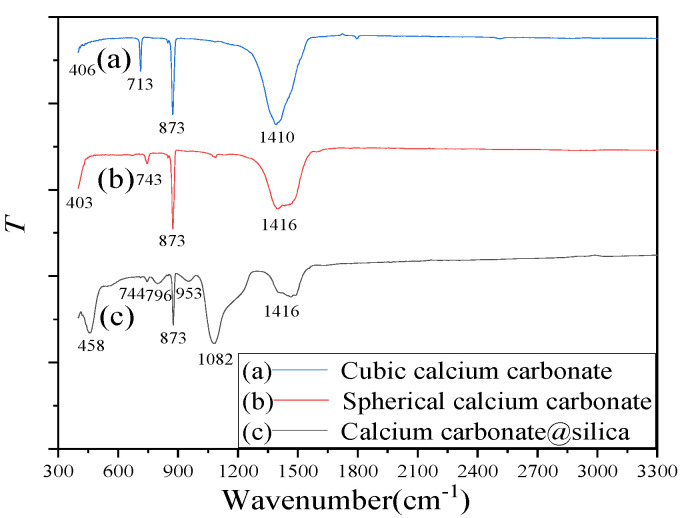
The FTIR of (a) cubic calcium carbonate, (b) spherical calcium carbonate, and (c) CaCO_3_@SiO_2_ composite material.

Figure 5 shows the infrared spectra of the cubic, spherical calcium carbonate and the composite material. The wavenumbers range change from 300 cm^−1^ to 2000 cm^−1^. It can be seen that calcium carbonates (a) and (b) have absorption peaks at 1410 cm^−1^, 1416 cm^−1^, 873 cm^−1^, 713 cm^−1^,a nd 743 cm^−1^, respectively. The absorption peaks at 1410 cm^−1^ and 1416 cm^−1^ are the C-O asymmetric stretching vibration [56]. The peak at 873 cm^−1^ is attributed to the out-of-plane bending vibration of the C-O bond [57]. For calcite and vaterite, the C-O asymmetric stretching vibration and out of plane bending vibrations, leading to the most intense peak, are at very similar positions, and thus cannot be used to distinguish between calcite and vaterite [58]. The peak at 713 cm^−1^ is attributed to the in-plane deformation vibration of the C-O bond of calcite crystal while the peak at 743 cm^−1^ is the characteristic absorption peaks of vaterite [59]. It can be seen that the composite material (c) has absorption peaks at 1416 cm^−1^, 1082 cm^−1^, 953 cm^−1^, 873 cm^−1^, 796 cm^−1^, 744 cm^−1^, and 458 cm^−1^, respectively. The characteristic peak at 1082 cm^−1^ is attributed to the stretching symmetric vibrations of Si–O–Si. The peak at 953 cm^−1^ belongs to the asymmetric vibration absorption peak of Si-OH [60]. The peak at 796 cm^−1^ and 458 cm^−1^ is the symmetric stretching vibration and bending vibration of Si-O bond [61].

**Figure 6:**
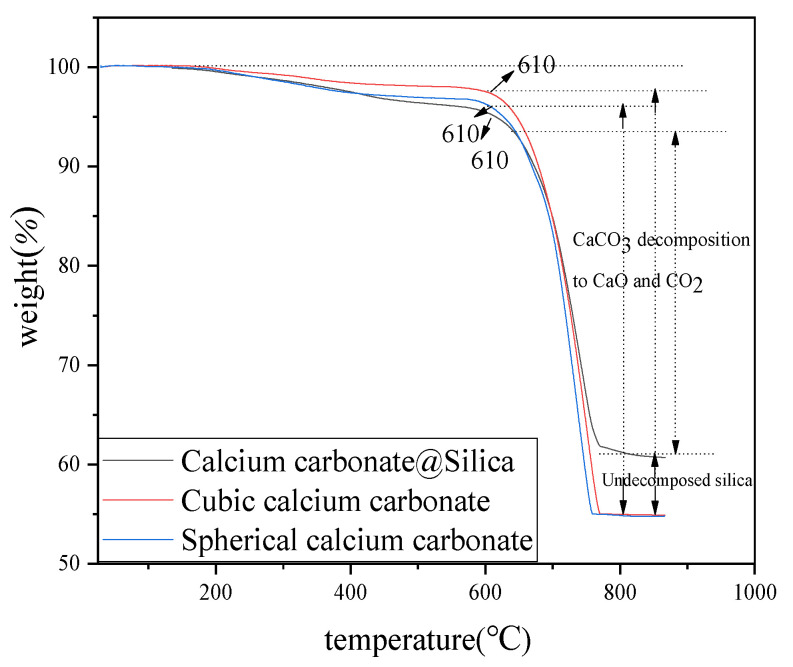
The TGA of CaCO_3_@SiO_2_ composite material and calcium carbonate. The temperature rises from 25 °C to 900 °C.

Thermogravimetry can be used to test the thermal stability of materials [62,63]. The TGA curves comparison between cubic calcium carbonate, spherical calcium carbonate and the composite material are shown in Figure 6. The weight loss of cubic and spherical calcium carbonate are mainly divided into two stages. The first region from room temperature 25 °C to 610 °C and the weight loss rate are about 2~4% and 3~5%. This is caused by intramolecular dehydration and carbonization of surfactants and cosurfactants adsorbed on the surface of calcium carbonate [64]. The weight loss rate is about 41.8~42.7% in the second region from temperature 610 °C to 790 °C, which is attributed to the thermal decomposition of calcium carbonate into calcium oxide and CO_2_, while CO_2_ escapes from the system. The weight loss of CaCO_3_@SiO_2_ is also mainly divided into two stages. At the first region of 25 °C~610 °C, the weight loss rate of CaCO_3_@SiO_2_ is about 5~6%, which is a little bit higher than cubic calcium carbonate and spherical calcium carbonate. This is because the composite material has a larger specific surface area, so it can adsorb more surfactants and cosurfactants, resulting in a larger weight loss rate of CaCO_3_@SiO_2_ in this stage. The second weight loss between 610 °C and 790 °C is due to part of the silica being undecomposed, so the weight loss rate is only about 34.8~35.7%. 

**Figure 7:**
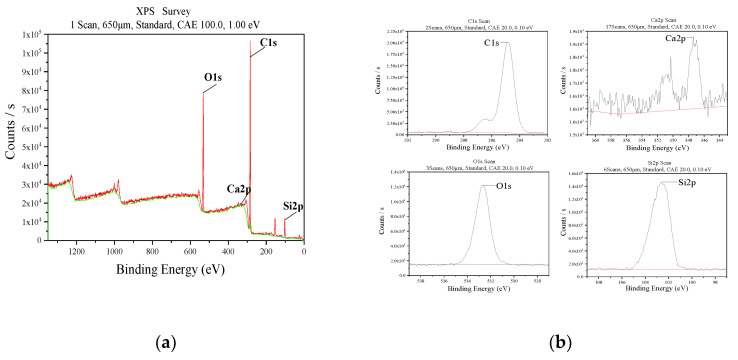
(**a**) The XPS spectra of CaCO_3_@SiO_2_ composite material. (**b**) The partial enlarged detail of (**a**).

Figure 7 shows the XPS characterization to verify that silica is loaded on the surface of the calcium carbonate. The peaks of O1s, Ca2p, C1s, and Si2p were shown by spectral scanning. The binding energies of C1s and O1s show characteristic peaks of the composite at 285.6 eV and 532.7 eV, respectively. The characteristic absorption peaks of Ca2p and Si2p appear at 347.5 eV and 101.5 eV, which correspond to the small figure in Figure 7, respectively [65]. The XPS spectrum of each element scan confirms that silica is successfully loaded on the surface of calcium carbonate.

### 3.3. Particle Size Analysis of Materials

**Figure 8:**
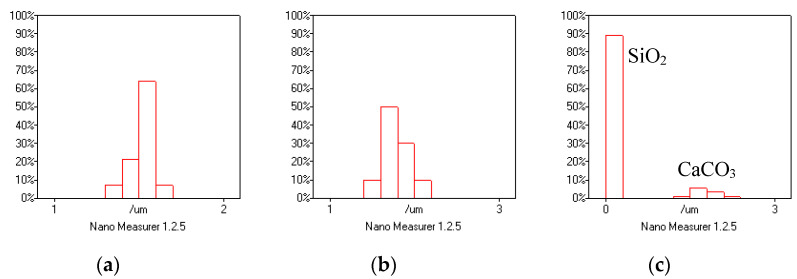
Particle size analysis of (**a**) cubic calcium carbonate, (**b**) spherical calcium carbonate, and (**c**) composite material.

Figure 8 shows the particle size analysis of the cubic, spherical calcium carbonate, and the composite material. The size and shape characterization of materials were based on SEM image analysis. The relevant length scale of cubic calcium carbonate is the side length. The spherical calcium carbonate and silica are approximately spherical and the relevant length scale is the diameter of spherical calcium carbonate and silica. In Figure 8c, the distribution of silica was shown on the left and the calcium carbonate was on the right. Nano measure software was used to analyze all images to obtain the mean values of length. Figure 8 shows the particle size distribution of materials. According to the particle size distribution, cubic calcium carbonate has a diameter of about 1.55 μm. The diameter of spherical calcium carbonate is about 1.80 μm. In the composite, the diameter of the large sphere is about 2.05 μm and the diameter of the spherical crown is about 0.25 μm. The standard deviation calculated by standard deviation formula are ±0.08, ±0.15, and ±0.25, respectively, as shown in Table 1.

### 3.4. Measurement of Hydrophobicity and Its Relationship with Roughness

Because the surface of CaCO_3_@SiO_2_ composite material and spherical calcium carbonate particles prepared in our lab were similar to the mastoid of lotus leaves surface due to the nano spherical silica adhere to the surface of the micron calcium carbonate, it will be interesting to see whether these materials will be superhydrophobic.

**Figure 9:**
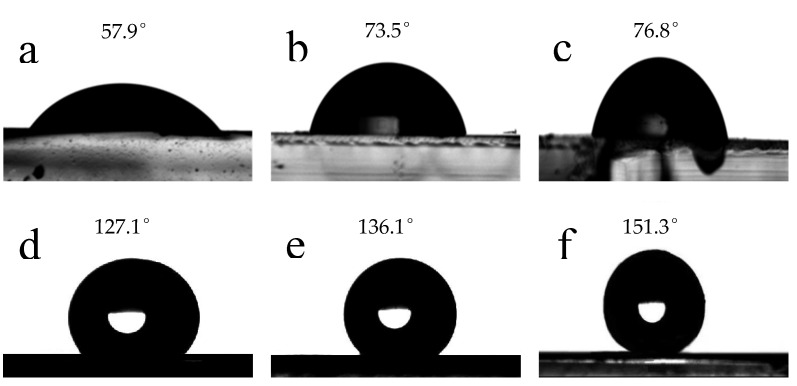
Contact angles of the materials coating on the glass. Unmodified: (**a**) Cubic calcium carbonate, (**b**) spherical calcium carbonate, (**c**) composite material. Modified by stearic acid: (**d**) cubic calcium carbonate, (**e**) spherical calcium carbonate, (**f**) composite material.

The hydrophobic performance was obtained by measuring the contact angle of water droplets on the surface of the materials [66,67]. The contact angles of the cubic calcium carbonate, spherical calcium carbonate and composite material are shown in Figure 9. The contact angle of cubic calcium carbonate, spherical calcium carbonate, and CaCO_3_@SiO_2_ composite material are 57.9°, 73.5°, and 76.8°, respectively.

In addition, the “lotus leaf” effect is also due to the combined action of its surface wax composition and micron-nano composite structure, which endows the lotus leaf with its unique superhydrophobicity and self-cleaning ability. Therefore, the hydrophobicity of the cubic calcium carbonate, spherical calcium carbonate, and composite material were further surface-treated by stearic acid, then made a coating on the glass. Their contact angles of the modified materials were measured. It can be seen from Figure 9d–f that the contact angles of the modified materials (*θ*_e_) were 127.1°, 136.1°, and 151.3°, respectively. It was found that the hydrophobic performance of these materials was improved significantly after being modified by stearic acid, in particular, the composite material could achieve the purpose of superhydrophobic. Because the three different materials were all coated by stearic acid, the difference of hydrophobicity is mainly caused by the difference of their surface structure.

According to the Cassie model theory, the different roughness also has a certain different hydrophobicity. As can be seen from Figure 3, the surface of cubic calcium carbonate particles is relatively smooth, while the morphology of spherical calcium carbonate and composite material shows irregular spherical crowns. The size of the spherical crowns significantly impacts the roughness of the material, resulting in different hydrophobicity of the material.

**Figure 10:**
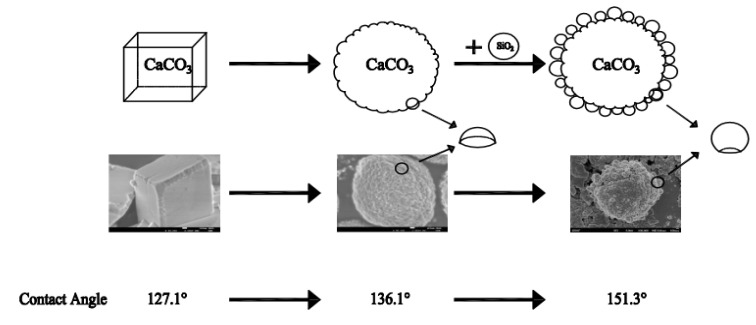
Calculation schematic diagram and experimental contact angle of different shapes of calcium carbonate. From cubic calcium carbonate to spherical calcium carbonate, then to composite material, the roughness of the materials is increasing.

As shown in Figure 10, spherical calcium carbonate and composite material can be regarded as micro spheres covered with nano spheres. The difference is that spherical calcium carbonate has less convex parts, while composite materials have more convex parts, which leads to the increase of roughness of materials. We assumed that the spherical crown of spherical calcium carbonate is 1/3 height of the nano spherical CaCO_3_ with a diameter of 60 nm, and the composite material is 2/3 height of the nano spherical SiO_2_ with a diameter of 400 nm. The specific surface area of spherical calcium carbonate and composite material can be calculated by calculating the area of the sphere crown. Because the surface of cubic calcium carbonate is flat, so it was used as a reference, according to the definition, the roughness *r* of the spherical calcium carbonate and CaCO_3_@SiO_2_ composite material surfaces are their specific surface area divided by the specific surface area of cubic calcium carbonate.

Assuming that the roughness of cubic calcium carbonate surface is 1, the apparent contact angle *θ*_r_ of spherical calcium carbonate and composite material calculated by the Cassie equation is shown in Table 2. Meanwhile, the standard deviation of water contact angle is also added in Table 2. The superhydrophobic needle used for measurement is 0.51 mm outside diameter, 0.25 mm inside diameter, 38.1 mm in length, and the amount of water in each injection is 2 μL per time. According to experimental results, the superhydrophobicity of the composite is stable, and the standard deviations of experimental contact angle are less than 1°, as shown in Table 2. The error of the calculation results (*θ*_r_) was also calculated from the error of the particle size, as shown in Table 2. From Table 2, we can also see that the results from calculation were consistent with those from experiment (*θ*_e_). According to the calculation, the roughness has a significant impact on the hydrophobicity, as the greater of the surface roughness, the better the hydrophobicity, which is consistent with the Cassie model. The material superhydrophobic can be modified by increasing the surface roughness.

## 4. Conclusions

In this paper, a new composite material of CaCO_3_@SiO_2_ was prepared by combining the nano structure with the micron structure, and the superhydrophobicity of the material was realized by increasing the surface roughness. The influence of the roughness on the hydrophobicity of the material was studied. It was found that after modification with stearic acid, the CaCO_3_@SiO_2_ composite had good superhydrophobicity, as the contact angle of CaCO_3_@SiO_2_ composite could reach 151.3°. Because all the composite materials were coated by stearic acid, their structure became the most important factor affecting hydrophobicity. The composite material we prepared achieves superhydrophobic performance due to the high roughness, which also provides a considerable prospect for us to prepare the superhydrophobic materials in the future. This study provides a new idea and method to study the superhydrophobic properties of rough surfaces by using the Cassie equation. The materials which we prepared are expected to be used as superhydrophobic coatings, for raincoats, waterproof glass, in the construction industry, and so on.

## Figures and Tables

**Table 1:** Average particle size and standard deviation of different materials.

	Cubic Calcium Carbonate	Spherical Calcium Carbonate	Composite Material
Mean value	1.55 μm	1.80 μm	2.05 μm
Standard deviation	±0.08	±0.15	±0.25

**Table 2:** Comparison between experiment and theoretical calculation.

	Cubic Calcium Carbonate	Spherical Calcium Carbonate	Composite Material
Roughness (*r*)	1	1.3	2.3
Experimental results (*θ*_e_)	127.1° ± 0.85°	136.1° ± 0.72°	151.3° ± 0.76°
Calculation results (*θ*_r_)	127.1° ± 1.65°	135.2° ± 2.20°	150.1° ± 2.55°

## Data Availability

Not applicable.
